# The severity of rat liver injury by fructose and high fat depends on the degree of respiratory dysfunction and oxidative stress induced in mitochondria

**DOI:** 10.1186/s12944-019-1024-5

**Published:** 2019-03-30

**Authors:** Claudia Isabel García-Berumen, Omar Ortiz-Avila, Manuel Alejandro Vargas-Vargas, Bricia A. del Rosario-Tamayo, Clotilde Guajardo-López, Alfredo Saavedra-Molina, Alain Raimundo Rodríguez-Orozco, Christian Cortés-Rojo

**Affiliations:** 1 Instituto de Investigaciones Químico-Biológicas, Universidad Michoacana de San Nicolás de Hidalgo, Edificio B-3, Ciudad Universitaria, 58030 Morelia, Michoacán Mexico; 2 Hospital General Regional 36, Instituto Mexicano del Seguro Social – IMSS, 72090 Puebla, Puebla Mexico; 3 Facultad de Ciencias Médicas y Biológicas “Dr. Ignacio Chávez”, Universidad Michoacana de San Nicolás de Hidalgo, 58020 Morelia, Michoacán Mexico; 4 Hospital Regional de Alta Especialidad del Instituto de Seguridad y Servicios Sociales de los Trabajadores del Estado – ISSSTE, Carr. Morelia-Atapaneo Km 6, Atapaneo, 58300 Morelia, Michoacán Mexico

**Keywords:** NAFLD, liver steatosis, Mitochondria, Respiratory chain, Complex I, lipid peroxidation

## Abstract

**Background:**

High fat or fructose induces non-alcoholic fatty liver disease (NAFLD) accompanied of mitochondrial dysfunction and oxidative stress. Controversy remains about whether fructose or fat is more deleterious for NAFLD development. To get more insights about this issue and to determine if the severity of liver disease induced by fructose or fat is related to degree of mitochondrial dysfunction, we compared the effects of diets containing high fat (HF), fructose (Fr) or high fat plus fructose (HF + Fr) on NAFLD development, mitochondrial function, ROS production and lipid peroxidation.

**Methods:**

Wistar rats were assigned to four groups: Control, fed with standard rodent chow; High fat (HF), supplemented with lard and hydrogenated vegetable oil; Fructose (Fr), supplemented with 25% fructose in the drinking water; High fat plus fructose group (HF + Fr), fed with both HF and Fr diets. Rats were sacrificed after 6 weeks of diets consumption and the liver was excised for histopathological analysis by hematoxylin and eosin staining and for mitochondria isolation. Mitochondrial function was evaluated by measuring both mitochondrial respiration and complex I activity. Lipid peroxidation and ROS production were evaluated in mitochondria by the thiobarbituric acid method and with the fluorescent ROS probe 2,4-H_2_DCFDA, respectively.

**Results:**

Fr group underwent the lower degree of both liver damage and mitochondrial dysfunction that manifested like less than 20% of hepatocytes with microvesicular steatosis and partial decrease in state 3 respiration, respectively. HF group displayed an intermediate degree of damage as it showed 40% of hepatocytes with microvesicular steatosis and diminution of both state 3 respiration and complex I activity. HF + Fr group displayed more severe damage as showed microvesicular steatosis in 60% of hepatocytes and inflammation, while mitochondria exhibited fully inhibited state 3 respiration, impaired complex I activity and increased ROS generation. Exacerbation of mitochondrial lipid peroxidation was observed in both the Fr and HF + Fr groups.

**Conclusion:**

Severity of liver injury induced by fructose or fat was related to the degree of dysfunction and oxidative damage in mitochondria. Attention should be paid on the serious effects observed in the HF + Fr group as the typical Western diet is rich in both fat and carbohydrates.

## Introduction

Non-alcoholic fatty liver disease (NAFLD) is defined as excessive hepatic lipid accumulation in individuals whose alcohol intake is not significant and without any other hepatic disease. NAFLD encompasses a spectrum of liver alterations ranging from non-alcoholic fatty liver (NAFL), when steatosis is observed in absence of hepatocyte ballooning, non-alcoholic steatohepatitis (NASH), when steatosis is accompanied of ballooning and inflammation, to cirrhosis [1]. NAFLD is one of the most prevalent liver diseases in the world [2], affecting one third of the population in developed countries and increasing overall liver-related morbidity and mortality [3]. High-fat diets were initially believed to be the primary driver of the obesity epidemic [4], which was associated with increasing NAFLD prevalence [5]. Consequently, fat was substituted by fructose because this carbohydrate, in contrast with glucose, does not stimulate insulin secretion. However, fructose leads to liver stress due to excessive phosphorylation of this carbohydrate at the expense of ATP, causing phosphate deficiency, AMP accumulation, and increased synthesis of both uric acid and triglycerides [6, 7].

Diets with excessive amounts of fructose or fat induce hepatic mitochondrial dysfunction. Fructose increases the supply of electrons to the electron transport chain (ETC) by upregulating the tricarboxylic acid cycle [8], this along with impaired complex IV activity, increased mitochondrial levels of ROS and lipid peroxidation [9]. On the other hand, excessive fat intake impairs mitochondrial respiration in the phosphorylating state (i.e. mitochondrial state 3 respiration), inhibits complex IV activity, increases lipid peroxidation and augments ROS generation [10, 11].

The central role of mitochondrial dysfunction in NAFLD progression has been revealed by its alleviation with mitochondria-targeted strategies. For example, the deletion of the lysocardiolipin acyltransferase ALCAT-1 ameliorated NAFLD by inhibiting cardiolipin remodeling and improving both mitochondrial respiration and oxidative stress [11]. Furthermore, the alleviation of NAFLD in rodents by mitochondria-targeted antioxidants has been attributed to decreased lipid peroxidation, attenuation of oxidative stress and the inhibition of apoptosis [12].

On the other hand, it has been shown that diets containing high fat plus fructose induce more damage in both liver and mitochondria than a diet containing only high fat [13]. However, the effects of fructose alone were not compared with the effects of high fat or high fat plus fructose. This is an important issue to address, because the role of fructose in the development of metabolic syndrome has been put in doubt, thus there is not yet a definitive consensus about whether fat or fructose is more detrimental for NAFLD progression [14, 15]. Furthermore, it is unknown whether the severity of liver disease is related to the degree of mitochondrial dysfunction caused by fructose or fat. To address these issues, we have compared the effects of diets enriched in fructose (Fr), high fat (HF) and high fat plus fructose (HF + Fr) on NAFLD development, dyslipidemia, mitochondrial function, ROS levels and lipid peroxidation.

## Materials and methods

### Animals and experimental groups

Male Wistar rats weighing 250–350 g were used in this study. Each rat was housed in individual cages and maintained at room temperature with day/night cycles of 12 h/12 h, with free access to diets and water or fructose. Animals were randomly assigned to four groups (Table 1): 1) control group: fed only with standard rodent chow; 2) fructose group (Fr): fed with standard rodent chow plus 25% fructose in the drinking water; 3) high-fat group (HF): fed with the HF diet; 4) high fat plus fructose group (HF + Fr): fed with the HF diet plus 25% fructose in the drinking water. Diets were provided for 6 weeks. Food intake was limited to 20 g daily per rat to avoid the rancidity of not immediately ingesting food in the groups whose diets contained high fat. 250 mL of water or fructose solution was given daily to each rat, except that fructose was given to the HF + Fr group every other day at the beginning of the 4th week, as its consumption decreased significantly at that time in this group. All the procedures with animals were performed according to the Federal Regulations for the Use and Care of Animals (NOM-062-ZOO-1999) issued by the Mexican Ministry of Agriculture.

**Table 1:** Experimental groups and preparation of diets

Diet components	Experimental groups			
	Control	HF	Fr	HF + Fr
Standard rodent chow^b^, %	100	47.5	100	47.5
Lard^c^, %		10.1		10.1
Hydrogenated vegetable oil^d^, %		40.6		40.6
Sodium cholate^e^, %		1.3		1.3
Choline chloride^e^, %		0.3		0.3
Thiouracil^e^, %		0.2		0.2
Fructose^f^, %*w*/*v*			25^a^	25^a^

Diets were provided during 6 weeks to Wistar rats with an initial weight of 250-350 g. ^a^Fructose was given in the drinking water. ^b^Laboratory Rodent Diet 5001, LabDiet, St. Louis, MO, USA. The composition of this diet can be consulted in [49]. ^c^JC Fortes, Empacadora San Benito, México. ^d^Manteca Inca, ACH Foods México, S. de R.L. de C.V. ^e^Sigma-Aldrich, St. Louis, MO, USA. ^f^Grupo Químico Contreras, S.A. de C.V. México

High-fat diet (HF) was prepared with 47.5% standard rodent chow (Laboratory Rodent Diet 5001, LabDiet, St. Louis, MO, USA), 10.1% lard, 40.6% hydrogenated vegetable oil, 1.3% sodium cholate, 0.3% choline chloride and 0.2% thiouracil (Table 1). Standard rodent chow contained 23.9% protein (28.5% of total calories), 5.0% fat (13.4% of total calories) and 48.7% carbohydrate (58.1% of total calories). HF diet contained 14.7% protein (8.8% of total calories), 53.8% fat (73.2% of total calories) and 29.8% carbohydrate (18.0% of total calories). Laboratory Rodent Diet 5001, which was used as the starting material to prepare the diets, exceeding several-fold the recommendations in vitamins and minerals made by the committee on AIN-93 purified diets for laboratory rodents [16], except for chromium, that is contained in similar concentrations in both diets, and for vitamin E, that is 1.78-fold higher in the AIN-93 diet than in the Laboratory Rodent Diet 5001.

### Isolation of mitochondria

At the end of the treatments, rats were fasted 14 h and sacrificed by decapitation. The liver was excised and placed into ice-cold isolation solution (Medium 1) containing 220 mM mannitol, 70 mM sucrose, 1 mM EGTA, and 2 mM MOPS (pH 7.4). The liver was cut, washed, and homogenized with a Potter-Elvehjem homogenizer. The homogenate was centrifuged at 314 x g. Subsequently, the supernatant was decanted and centrifuged at 4410 x g. The resulting pellet was washed with a solution (Medium 2) containing 220 mM mannitol, 70 mM sucrose, and 2 mM MOPS (pH 7.4), and centrifuged at 6350 x g. Finally, the pellet was re-suspended in 500 μl of medium 2. Each centrifugation was performed during 10 min at 4 °C [17]. Mitochondrial protein concentration was measured by the Biuret method.

### Determination of biochemical parameters in serum and weight gain

At the end of the six-weeks of treatments with diets, rats were fasted for 14 h and blood was recollected after the sacrifice for obtaining serum. Glucose, total cholesterol (TC), and triglyceride (TG) levels were measured by enzymatic methods with kits from VITROS Chemistry Products (Ortho Clinical Diagnostics Inc. Rochester, NY, USA), according to the manufacturer’s instructions. Weight gain was determined by subtracting the weight of the animals after the six-weeks of treatments, measured right before sacrifice, minus the weight at the beginning of the treatments.

### Histological analyses of livers

Small sections of the livers, obtained at the sacrifice of animals, were fixed in 10% formalin, embedded in paraffin blocks, sectioned (5 μm thick) and stained with hematoxylin and eosin. Light microscopy was used for evaluation of steatosis, inflammation and hepatocyte ballooning. Microvesicular steatosis was evaluated like cytosolic accumulation of little lipid droplets not perturbing the central location of the nucleus. Macrovesicular steatosis was evaluated like cytosolic presence of large lipid drops that move the nucleus from its central position into the cell periphery [18].

### Evaluation of mitochondrial respiration

Mitochondrial respiration was measured in basal, oligomycin-induced, state 4 (state 4_O_) and phosphorylating state (state 3) by determining the oxygen consumption rate of freshly isolated mitochondria using a Clark-type electrode coupled to a YSI 5300A biological oxygen monitor and connected to a computer for data acquisition. 1.25 mg of mitochondrial protein was placed into a sealed glass chamber containing respiratory buffer with 100 mM KCl, 10 mM HEPES, 3 mM KH_2_PO_4_ and 3 mM MgCl_2_ (pH 7.4). The final volume was adjusted to 2.5 mL. Respiration traces were started after adding mitochondria and 10 mM glutamate/malate as respiratory substrate. State 3 was stimulated with 0.2 mM ADP. State 4_O_ was induced by adding 1.4 μg/mL oligomycin. Respiratory control ratio (RCR) was calculated by dividing the respiration rate in state 3 vs the respiration rate in state 4_O_.

### Determination of complex I activity

0.1 mg/mL of mitochondrial protein were resuspended in a buffer with 50 mM KH_2_PO_4_ (pH 6.9) and incubated by 5 min with 1 μg antimycin A and 1 mM KCN in a final volume of 1 mL. Then, 5 mM K_3_Fe(CN)_6_ was added as electron acceptor and absorbance was registered at 340 nm in a Shimadzu UV2550 spectrophotometer. After 1 min, NADH was added and its oxidation was monitored during 4 min. The rate of NADH oxidation was calculated using the molar extinction coefficient of 16.3 mM^− 1^ cm^− 1^ for NADH and the slopes of the time-traces of NADH oxidation.

### Measurement of ROS levels

ROS levels were determined by measuring the oxidation of the fluorescent probe 2′,7′-dichlorodihydrofluorescein diacetate (H_2_DCFDA). 0.5 mg/mL of intact mitochondria and 1.25 mM H_2_DCFDA were incubated in a solution with 100 mM KCl, 10 mM HEPES, 3 mM MgCl_2_ and 3 mM KH_2_PO_4_ (pH 7.4) during 20 min at 4 ^∘^C under constant stirring. Then, mitochondrial suspension was placed into a quartz cuvette and basal fluorescence was recorded over time. After 1 min, 10 mM glutamate/malate was added as substrate for the ETC and the changes in H_2_DCFDA fluorescence were further monitored by 15 min. Fluorescence changes were detected in a RF-5301PC spectrofluorophotometer (Shimadzu Corporation, Kyoto, Japan) (*λ*_ex_ 491 nm; *λ*_em_ 518 nm). ROS levels were calculated by subtracting the fluorescence (ΔF) detected after 15 min of substrate addition minus the fluorescence detected when substrate was added. ΔF was divided then by the milligrams of mitochondrial protein used in the assay.

### Lipid peroxidation assay

Lipid peroxidation was evaluated with the thiobarbituric acid (TBA) method [19]. Mitochondrial pellets (0.1 mg/mL protein) were washed twice and resuspended with 50 mM KH_2_PO_4_ buffer (pH 7.6) immediately before TBA assay to avoid false positive results due to the interaction of thiobarbituric acid with the carbohydrates present in mitochondria isolation buffers. Lipid peroxidation levels were reported as thiobarbituric acid reactive substances (TBARS) per milligram of mitochondrial protein.

### Data analysis

Data are expressed as mean ± standard error of the mean. Statistical differences of data were determined with two-way analysis of variance (ANOVA), followed by multiple comparisons analysis performed with post hoc Tukey test. Statistical significance was set at *P* < 0.05. Analysis were done with Sigma Plot 11.0 software (Systat Software, Inc., San Jose, CA, USA).

## Results

### Effects of fructose and fat on physiological parameters

**Fig. 1:**
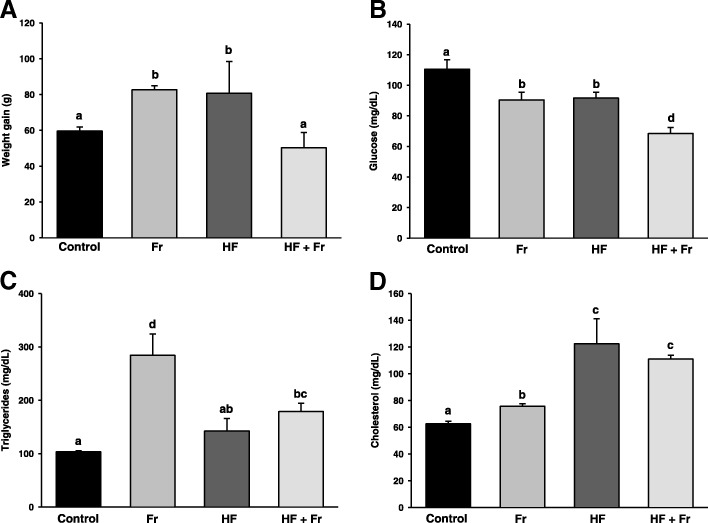
Weight gain (**a**) fasting serum glucose (**b**), fasting serum triglycerides (**c**) and fasting serum cholesterol (**d**) in rats that were fed for 6 weeks with diets containing normal rodent chow (Control), fructose (Fr), high fat (HF), and high fat plus fructose (HF + Fr). Weight gain was determined by subtracting the weight of the animals after six-weeks treatments with the diets, determined right before sacrifice, minus the weight at the beginning of the treatments. The results are presented as the mean ± S.E. of *n* ≥ 4. Different letters indicate statistically significant differences at *P* < 0.05

Control group showed a weight gain at the end of the study of 59.6 g (Fig. 1a). Higher weight gains of 82.7 and 80.7 g were observed in both Fr and HF groups, respectively. In contrast, there were no differences in weight gains between control and HF + Fr groups. Regarding food consumption, there were no differences in this parameter among all the groups, since the animals fully consumed the amount (20 g) of food that was provided daily. No differences in water or fructose intake were detected within the groups, as all the rats drank the 250 mL that were given daily, except for the HF + Fr group, which started to drink ~ 110 mL fructose at the beginning of the 4th week of treatment.

Serum glucose concentration of the control group was 110 mg/dL (Fig. 1b) and lower levels were detected in the Fr, HF and HF + Fr groups, with values of 90.4, 91.6, and 68.5 mg/dL, respectively. Serum triglycerides levels are shown in the Fig. 1c. In comparison to control group, triglycerides levels were 2.7- and 1.7-fold higher in both Fr and HF + Fr groups, respectively, while no significant changes were observed in the HF group. Serum cholesterol levels (Fig. 1d) augmented moderately in the Fr group when compared to the control group. In contrast, cholesterol levels incremented up to 1.9 - and 1.7 - fold in the HF and HF + Fr groups, respectively.

### Effects of fructose and fat on liver histology

**Fig. 2:**
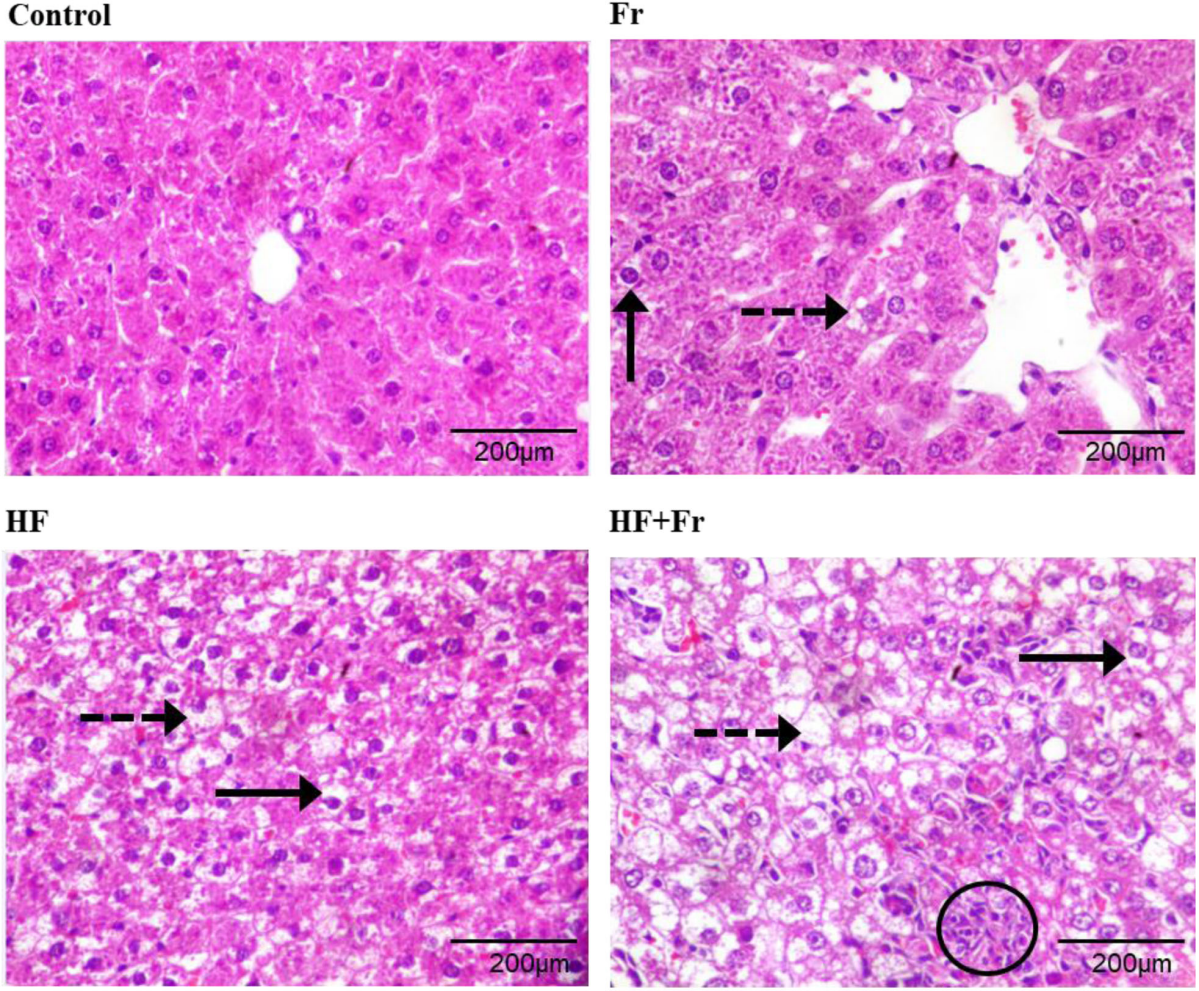
Histological examination of livers from rats that were fed for 6 weeks with diets containing normal rodent chow (Control), fructose (Fr), high fat (HF) and high fat plus fructose (HF + Fr). Black arrows indicate the presence of macrovesicular steatosis; black dotted arrows indicate the presence of microvesicular steatosis; circle indicates inflammatory infiltrate. Liver sections were stained with hematoxylin-eosin and pictures were taken at 40X magnification

Liver histological examination (Fig. 2 and Table 2) shows that livers from the Fr group displayed a lower percentage of hepatocytes with macrovesicular (10–20%, black lines) and microvesicular (15%, dotted arrows) steatosis, followed by the HF group with 40% of hepatocytes with microvesicular steatosis and 60% with macrovesicular steatosis. The highest degree of steatosis was observed in the HF + Fr group, with 60% of hepatocytes with microvesicular steatosis and 80% with macrovesicular steatosis (in some cases, hepatocytes displayed both forms of steatosis). Furthermore, the percentage of ballooned cells was 60% for both Fr and HF groups and 70% for the HF + Fr group. (Fig. 2). In addition, the HF + Fr group also showed chronic inflammatory infiltrate (circle).

**Table 2:** Quantification of histological alterations induced in the livers of fructose (Fr), high fat (HF) and high fat plus fructose (HF + Fr) groups

Liver alterations	Experimental groups			
	Control	Fr	HF	HF + Fr
Microvesicular steatosis	1%	10–20%	40%	60%
Macrovesicular steatosis	N.D.	15%	60%	80%
Ballooning	N.D.	60%	60%	70%

Data are expressed as the percentage of hepatocytes showing each type of alteration

### Effects of fructose and fat on mitochondrial function

**Fig. 3:**
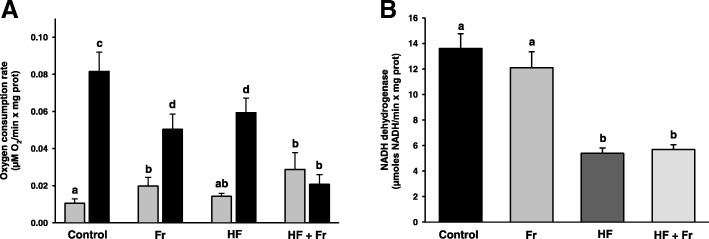
Rate of respiration (**a**) and complex I activity (**b**) of liver mitochondria from rats that were fed for six weeks with diets containing normal rodent chow (Control), fructose (Fr), high fat (HF) and high fat plus fructose (HF + Fr). Mitochondria were fueled with glutamate – malate. Respiration was measured in state 3 (black bars) and in oligomycin-induced state 4 (gray bars). The results are presented as the mean ± S.E. of *n* ≥ 3. Different letters indicate statistically significant differences at *P* < 0.05

In mitochondria from the control group, the respiration rate was ~ 7.7-fold higher in state 3 than in state 4_O_, thus resulting in a respiratory control ratio (RCR) of 7.7 (Fig. 3a). Respiration rate in state 3 decreased in the Fr and HF groups 1.6- and 1.4- fold, respectively, in comparison to the control group. This resulted in RCR values of 2.5 and 4.1, respectively. A 1.8-fold increase in state 4 respiration also contributed to the lower RCR observed in the Fr group. Rate of respiration in state 3 decreased 3.9-fold in the HF + Fr group when compared to the control group, while state 4 respiration increased 2.7-fold, which resulted in a RCR of 0.7. On the other hand, complex I activity decreased ~ 2.3-fold in mitochondria for both the HF and the HF + Fr groups (Fig. 3b). In contrast, the activity in the Fr group remained unaltered.

### Effects of fructose and fat on mitochondrial ROS generation and lipid peroxidation

**Fig. 4:**
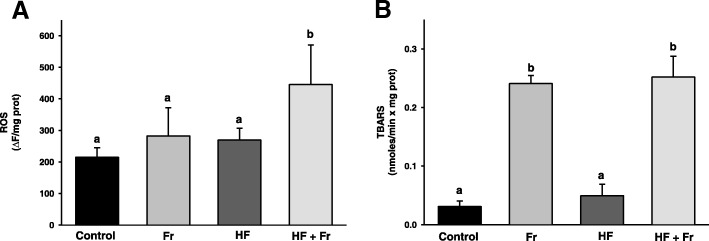
Levels of ROS (**a**) and lipid peroxidation (**b**) of liver mitochondria from rats that were fed for six weeks with diets containing normal rodent chow (control), fructose (Fr), high fat (HF) and high fat plus fructose (HF + Fr). The results are presented as the mean ± S.E. of n ≥ 3. Different letters indicate statistically significant differences at *P* < 0.05

Mitochondrial ROS levels doubled in the HF + Fr group in comparison to the control group (Fig. 4a). In contrast, no changes in the ROS levels were found in mitochondria for both the Fr and the HF groups. The levels of mitochondrial lipid peroxidation are presented in the Fig. 4b. In comparison to mitochondria of the control group, the levels of lipid peroxidation increased ~ 8 times in mitochondria from both the Fr and the HF + Fr groups, while no changes were detected in the HF group.

## Discussion

Inclusion of fructose and/or fat in the diet induced a variable degree of hepatic damage. The HF + Fr group exhibited the most negative outcome, as evidenced by the higher percentage of hepatocytes with microvesicular and macrovesicular steatosis and inflammation. The HF group showed an intermediate presence of steatosis; while the lowest percentage of hepatocytes with both types of steatosis was observed in the Fr group (Fig. 2 and Table 2).

As shown in Fig. 2, deleterious effects of fat were aggravated by fructose, since the HF group exhibited a lower extent of steatosis than the HF + Fr group (Fig. 2 and Table 2), besides the latter group also exhibited inflammation. In this regard, it has been hypothesized that the severity of liver damage correlates with the degree of oxidative stress in hepatocytes [20]. Accordingly, it was observed that the consumption of fat did not increase lipid peroxidation per se, as can be seen in the HF group (Fig. 4b). In contrast, fructose alone or combined with fat increased several-fold the levels of lipid peroxidation. Lipid peroxidation has been identified as an elicitor of NASH by triggering signaling cascades that mediates inflammation via an augmentation in the levels of malondialdehyde (MDA) and 4-hydroxynonenal (4-HNE), the end-products of lipid peroxidation [21, 22]. This suggest that lipid peroxidation induced by fructose might be triggering inflammation only when a higher degree of steatosis is established by high fat intake. Nevertheless, it cannot be ruled out that an increase of lipid peroxidation and inflammation might occurs by the consumption of a HF diet over a longer period time. Despite the Fr group exhibited high levels of mitochondrial lipid peroxidation, it also showed a low level of steatosis without changes in ROS production, which suggest that mitochondrial lipid peroxidation per se does not cause severe liver damage in the absence of other factors such as significant accumulation of fat or increased mitochondrial ROS production as was only observed in the HF + Fr group (Figs. 1 and 4a).

Another factor that may explain the presence of inflammation in the HF + Fr group is the pronounced mitochondrial dysfunction resulting from a sharp decrease in state 3 respiration yielding a RCR of 0.7 (Fig. 3a), which can be interpreted as a full impairment of oxidative phosphorylation. ATP depletion due to both augmented fructose metabolism [7] and impaired oxidative phosphorylation (Fig. 3a) might lead to cell death by necrosis in livers of the HF + Fr group, as ATP depletion is a hallmark of necrosis [23]. In turn, liver necrosis drives the release of intracellular content and activation of macrophages and neutrophils [24], which would be in concordance with the presence of inflammatory infiltrate seen in the HF + Fr group (Fig. 2).

A relationship was not found between the levels of lipid peroxidation and ROS, as lipid peroxidation increases in both the Fr and the HF + Fr groups, but ROS levels increases only in the latter one (Fig. 4). The absence of an apparent correlation among ROS and lipid peroxidation suggest that other factors different from ROS generation were responsible for increased lipid peroxidation by fructose. In this regard, induction of lipid peroxidation in liver by fructose has been associated to depletion of antioxidant defenses [25]. Lipid peroxidation is counteracted in mitochondria by phospholipid hydroperoxide glutathione peroxidase 4 (GPx4) using reduced glutathione (GSH) as an electron donor [26]. One catalytic round of GPx4 produces a molecule of oxidized glutathione (GSSG) that it is reduced back to two molecules of GSH by glutathione reductase (GR). It has been found that methylglyoxal (MGO), a reactive dicarbonyl involved in the production of advanced glycation end-products [27], inhibits the activities of GPx and GR [28]. Thus, a possible explanation for the several-fold increase of lipid peroxidation observed exclusively in the groups consuming fructose is that GPx4 and GR becomes inactivated by MGO produced during the hepatic metabolism of fructose.

Higher levels of ROS observed in mitochondria from the HF + Fr group (Fig. 4a) correlates well with null stimulation of respiration by ADP that yielded an RCR of 0.7 (Fig. 3a). Decreased respiration due to inhibition of ATP production by F_1_F_0_-ATP synthase leads to high rates of ROS production [29]. The lack of response to ADP addition can be interpreted like an impairment of F_1_F_0_-ATP synthase to produce ATP, which leads to decreased electron transfer and increased ROS generation. Furthermore, the partial decline of state 3 respiration observed in both the HF and the Fr groups, and therefore, their RCR of 4.1 and 2.5, respectively, is in concordance with their lower levels of ROS with respect to the HF + Fr group. Overall, these data suggest that the main factor increasing ROS production was the full impairment of state 3 respiration, although it cannot be discarded that severe impairment in the activity of antioxidants systems (e.g. glutathione system) may also account for the higher ROS levels observed in the HF + Fr group.

Low rates of mitochondrial respiration in the liver increase the NADH/NAD^+^ ratio [30], which leads to defective fatty acid β-oxidation and hepatic accumulation of triglycerides [31, 32]. This could explain exacerbated steatosis in the HF + Fr group (Fig. 2) as mitochondria from this group displayed the lower rate of state 3 respiration (Fig. 3a). Conversely, the lower severity of steatosis observed in the HF and Fr groups agree with their higher rates of state 3 respiration. On the other hand, complex I activity was lower in the HF group than in the Fr group (Fig. 3b); this might be involved in the higher degree of steatosis observed in the HF group by virtue of the role of complex I in NADH re-oxidation (i.e., the lower the complex I activity, the higher the NADH levels) and the dependence of fatty acid catabolism on low NADH/NAD^+^ ratios. On this basis, it can be hypothesized that higher accumulation of fat in the livers of HF group was due to a low rate of NADH re-oxidation caused by a highly decreased complex I activity, while the Fr group had a low hepatic fat accumulation thanks to a fully functional complex I. In summary, there is an inverse relationship between complex I activity, the rate of state 3 respiration, and steatosis severity, which might be related to modulation of fatty acid β-oxidation rate exerted by NADH/NAD^+^ ratio and with the role of complex I activity on NADH redox turnover.

It has been considered that macrovesicular steatosis has a good prognosis when presented alone, with rare progression to NASH or cirrhosis. Microvesicular steatosis, by the contrary, is a less benign entity than macrovesicular steatosis because it has a serious prognosis and it is associated to impaired β-oxidation [33]. Taking into account the association between impaired β-oxidation and the presence of microvesicular steatosis, it can be postulated that excessive fat deposition in the HF + Fr group might be largely due to the inability of mitochondria to re-oxidize NADH molecules produced during the β-oxidation, because low complex I activity in conjunction with fully inhibited oxidative phosphorylation. In the case of the HF group, intermediate fat deposition would be the result of low complex I activity along with partially functional oxidative phosphorylation, which may allow to re-oxidize NADH at intermediate levels. Finally, the relatively low deposition of fat in the Fr group would be attributed to higher capacity for NADH oxidation than in the other groups due to unaffected complex I activity and partially functional oxidative phosphorylation.

The pronounced deleterious effects of the HF + Fr diet are not trivial since the typical Western diet is abundant in high fat and high carbohydrates [34]. Besides, our results are consistent with another study showing that a high fat diet was significantly less deleterious than a high fat plus fructose diet, as the former induced only steatosis while the latter caused NASH. Furthermore, it was concluded that fructose was responsible for NASH development as the knockdown of the fructokinase gene prevented inflammation and fibrosis [35], which is also in line with our suggestion that fructose worsens the effects of fat by inducing necrotic cell death and inflammation. In contrast, it was shown in another report, that in comparison to our results, a high fat plus fructose diet had discrete effects on both respiration and lipid peroxidation in rat liver mitochondria [13], besides there were no differences between the effects produced by a high fat diet and the high fat plus fructose diet on these parameters. However, it must be stressed that in that work, diets were supplied for a shorter time (2 weeks) in comparison to this study (6 weeks), which may explain these different outcomes.

Several studies have shown that fructose does not affect weight gain [36–38], while others agree with our finding of increased weight by fructose [39–41]. Tillman et al. [38] have proposed that differences in weight response to fructose might be attributed to the way fructose was provided to animals (i.e. liquid or solid). For example, fructose was administered like aqueous solutions with high fructose corn syrup (HFCS) [39] or sucrose [41] in studies where fructose induced positive weight gain. In contrast, pelleted diet containing 60% fructose was given in studies showing no alteration on body mass [36–38]. Nevertheless, in one of these studies where weight gain was observed, sucrose was given in solid form [40]. Thus, there is no clear relation between the physical form in which fructose is administered and its effects on weight gain. Another possibility might be the different effects that fructose-containing carbohydrates may have in anorexigenic hormones like leptin. Fructose promotes weight gain by decreasing leptin blood levels and dysregulating its actions in energy balance [42]. On the contrary, it would be expected that leptin actions on body weight were not so altered with the HFCS or sucrose-containing diets, since these carbohydrates also contains glucose, which may counteract the effects of fructose on leptin secretion by inducing insulin secretion. However, this explanation does not fit with the outcomes of the studies referred above, since sucrose or HFCS was given in the studies where increased weight gain occurred [39–41]. In contrast, fructose was administered in the studies where no differences in weight gain were observed [36–38], which conflicts with our finding about weigh gain with fructose (Fig. 1a). Thus, the possibility remains that Wistar rats, the strain used in this study, present a different phenotypical response to fructose ingestion with respect to body mass, which guarantee further research to compare the effects of HFCS, sucrose or fructose, given in aqueous solution or pelleted, on the body mass of rat strains with different genetic backgrounds.

It was unexpected that there were no differences in weight gain between the HF + Fr and the control groups, and that even weight gain of the HF + Fr group was lower than that observed in both the HF and Fr groups (Fig. 1a). The most likely explanation for this finding is that fructose intake decreased more than 50% in the HF + Fr group at the beginning of the 4th week of treatment, which would be limiting calorie intake, and hence body weight gain. On the other hand, it can be hypothesized that lower weight gain in the HF + Fr group with respect to both the HF and the Fr groups may be due to combined result of exacerbated liver damage, highly impaired mitochondrial function, and a probable hyperinsulinemic status in rats of the HF + Fr group. Fructose is known to induce hyperinsulinemia [42]. Hyperinsulinemia in turn stimulates lipolysis in adipocytes and the release of free fatty acids into the bloodstream. Once in the liver, fatty acids are incorporated into triglycerides for their exportation in VLDL particles. Nevertheless, hyperinsulinemia also inhibits VLDL exportation to adipocytes [43], which may contribute in conjunction with increased lipolysis in adipocytes to decrease peripheric adiposity, as well to reduce fasting triglyceride levels as was observed in the HF + Fr group with respect to the Fr group (Fig. 1c). Probable hyperinsulinemic status might promote de novo triglyceride synthesis [44]; however, the deep impairment of oxidative phosphorylation in the HF + Fr group (Fig. 3a), and hence, the consequent failure in ATP synthesis, may counteract lipogenesis as this process is highly dependent on ATP [45], in this way leading to low availability of triglycerides to be exported to adipose tissue via VLDL. Probable inhibition of triglyceride synthesis also agrees with the severe degree of liver damage and higher levels of ROS observed in the HF + Fr group (Figs. 2 and 4a, respectively), as hepatic accumulation of free fatty acids due to inhibited lipogenesis produces oxidative stress and liver damage [44]. It may be argued against this hypothesis that the Fr group would have also exhibited a similar phenotype of decreased weight gain due to fructose-induced hyperinsulinemia. However, the different outcomes observed among the HF + Fr and the Fr groups for the levels of glucose and triglycerides, and in weight gain suggest important differences between them in hormonal metabolic regulation. The idea that the HF + Fr diet induces lower gain weight associated to hyperinsulinemia is supported by another study where reduction of weight gain, hyperinsulinemia and severe liver damage was found in rats fed with a HF + Fr + ethanol diet [46], although this comparison must be taken with caution, as the degree at which ethanol contributes to this phenotype has not been elucidated.

Regarding the impact of diets on blood lipids, the more prominent effect was the increase in triglycerides in the Fr group (Fig. 1c), which agrees with the hyperlipidemic effect of this carbohydrate due to stimulation of de novo lipogenesis [47]. Unexpectedly, serum triglycerides in the HF + Fr group were 1.6-fold lower than in the Fr group. A possible explanation for this observation is that the inhibition of oxidative phosphorylation in the HF + Fr group (Fig. 3a) might be decreasing the rate of the de novo lipogenesis since the latter process is highly dependent on ATP produced by mitochondrial respiration [45]. On the other hand, the hypercholesterolemic effect of the diets containing HF (Fig. 1d) was not surprising due to the high content of cholesterol in the lard used for diets preparation.

A limitation of this study is that we did not determine if the severity of NAFLD induced by high fat or fructose is dependent on differential expression of pro-inflammatory cytokines like IL-6 or TNFα, which have been involved in NAFLD pathogenesis [48]. Likewise, we did not analyze the impact of the mitochondrial alterations observed with each diet on cytokine expression profile. Another limitation is that we did not examine the impact over NAFLD progression of inhibiting lipid peroxidation, ROS generation, or of counteract the impairment of oxidative phosphorylation. All that information would be useful to establish a causal link between the alterations induced by high fat or fructose in mitochondrial function and the progression of NAFLD. Another limitation is the lack of data about the NADH/NAD^+^ ratio, the hepatic triglyceride content, and the rate of mitochondrial β-oxidation, which would allow to verify the negative influence of fructose or fat in the mitochondrial utilization of fatty acids and its relationship with the severity of steatosis. Finally, we did not analyze the effects of fructose and high fat for longer time periods.

## Conclusions

Despite producing the higher levels of lipid peroxidation, fructose provoked the less deleterious effects on both mitochondrial function and NAFLD since this carbohydrate partially decreased oxidative phosphorylation and induced the lower percentage of microvesicular steatosis. High fat exerted intermediate effects that manifested as decreasing in both oxidative phosphorylation and complex I activity in mitochondria, and intermediate levels of steatosis. The combination of high fat plus fructose produced the more deleterious effects in mitochondria including lipid peroxidation, enhanced ROS production, decreased complex I activity, and the full inhibition of oxidative phosphorylation, which fits well with the more severe liver damage induced by this diet that manifested as high levels of steatosis and inflammation. The latter might have implications for development of innovative strategies against NAFLD as the Western diet involves the simultaneous intake of excessive fructose and high fat. Hence, therapeutic approaches should be focusing in counteracting lipid peroxidation, excessive ROS production, and enhancing both oxidative phosphorylation and complex I activity.

## Abbreviations

2,4-H_2_DCFDA: 2′,7′-dichlorodihydrofluorescein diacetate
4-HNE: 4-hydroxynonenal
ALCAT-1: acyl-CoA:lysocardiolipin acyltransferase 1
ANOVA: analysis of variance
EGTA: ethylene glycol-bis(2-aminoethylether)-N,N,N′,N′-tetraacetic acid
ETC: electron transport chain
Fr: fructose
GPx4: phospholipid hydroperoxide glutathione peroxidase 4
GR: glutathione reductase
GSH: reduced glutathione
GSSG: oxidized glutathione
HEPES: 4-(2-hydroxyethyl)-1-piperazineethanesulfonic acid
HF + Fr: high fat plus fructose
HF: high fat
MDA: malondialdehyde
MGO: methylglyoxal
MOPS: 3-(N-Morpholino)propanesulfonic acid
NAFLD: non-alcoholic fatty liver disease
NASH: non-alcoholic steatohepatitis
RCR: respiratory control ratio
ROS: reactive oxygen species
TBA: thiobarbituric acid
TBARS: thiobarbituric acid reactive substances
TC: total cholesterol
TG: triglycerides
